# LncRNA H19: a novel player in the regulation of diabetic kidney disease

**DOI:** 10.3389/fendo.2023.1238981

**Published:** 2023-10-27

**Authors:** Qinrui Wu, Fengjuan Huang

**Affiliations:** Department of Endocrinology and Metabolism, The First Affiliated Hospital of Zhengzhou University, Zhengzhou, China

**Keywords:** LncRNA H19, diabetic kidney disease (DKD), pathogenesis, pathological changes, kidney cells

## Abstract

Diabetic kidney disease (DKD), one of the most severe complications of diabetes mellitus (DM), has received considerable attention owing to its increasing prevalence and contribution to chronic kidney disease (CKD) and end-stage kidney disease (ESRD). However, the use of drugs targeting DKD remains limited. Recent data suggest that long non-coding RNAs (lncRNAs) play a vital role in the development of DKD. The lncRNA H19 is the first imprinted gene, which is expressed in the embryo and down-regulated at birth, and its role in tumors has long been a subject of controversy, however, in recent years, it has received increasing attention in kidney disease. The LncRNA H19 is engaged in the pathological progression of DKD, including glomerulosclerosis and tubulointerstitial fibrosis via the induction of inflammatory responses, apoptosis, ferroptosis, pyroptosis, autophagy, and oxidative damage. In this review, we highlight the most recent research on the molecular mechanism and regulatory forms of lncRNA H19 in DKD, including epigenetic, post-transcriptional, and post-translational regulation, providing a new predictive marker and therapeutic target for the management of DKD.

## 1 Introduction

Diabetes mellitus (DM) is a heterogeneous group of disorders of glucose metabolism dysfunction (1), the global prevalence of which has risen dramatically in recent years, a figure projected to reach 783 million by 2045 (2). Excessive exposure to fluctuating glucose concentrations is increasingly recognized as an important pathological factor contributing to diabetic microvascular complications, which eventually lead to diabetic kidney disease, retinopathy, and neuropathy (3, 4). Among these, diabetic kidney disease (DKD) is of great concerns due to its high prevalence and harmful effects on the kidney. It is estimated that more than 40% of individuals with diabetes worldwide are affected by DKD, which is a leading cause of chronic kidney disease (CKD) and end-stage kidney disease (ESRD) (5). Moreover, DKD patients with ESRD have an alarmingly high mortality rate (6), and the therapy typically required for survival is renal replacement therapy (RRT), such as kidney dialysis or transplantation, both of which impose a huge financial burden on individuals and society (7). Hence, a deeper understanding of the molecular mechanisms underlying DKD is urgently required to assist in the discovery of effective therapeutics that can halt its progression and lower the associated risks.

Hyperglycemia is widely considered the primary etiological factor in the development of DKD, as it encourages metabolic and hemodynamic changes in the kidney, causing alterations in renal component structure and function such as glomerulosclerosis, tubulointerstitial inflammation, and fibrosis, which contribute to a decrease in glomerular filtration rate (GFR), ever-increasing albuminuria, and ultimately renal failure (8–10). Additionally, accumulating data indicate that the pathophysiology of DKD may be influenced by oxidative stress (OS), hypoxia, and overactivity of the renin-angiotensin-aldosterone system (RAAS) (11). Moreover, the available data suggest that long non-coding RNAs (lncRNAs) may be involved in the development of DKD, offering a novel avenue for the DKD therapy (12).

Long non-coding RNAs (LncRNAs), which are linear transcripts longer than 200 nucleotides that lack protein-translation potential, are of low abundance and stability making up the majority of non-coding RNAs (ncRNAs) in the genome (13, 14). Typically, lncRNAs are classified into multiple subtypes based on their positions, including sense, antisense, intronic, bidirectional, and intergenic (15). They exert biological functions by modulating various physiological processes, including cell differentiation, proliferation, and responses to various stresses (16). Recently, an expanding collection of studies have demonstrated differential expression of lncRNAs at the cellular and tissue levels in the kidney, ultimately leading to damage to cell masses that accelerate renal fibrosis and glomerulosclerosis (17).

**Figure 1:**
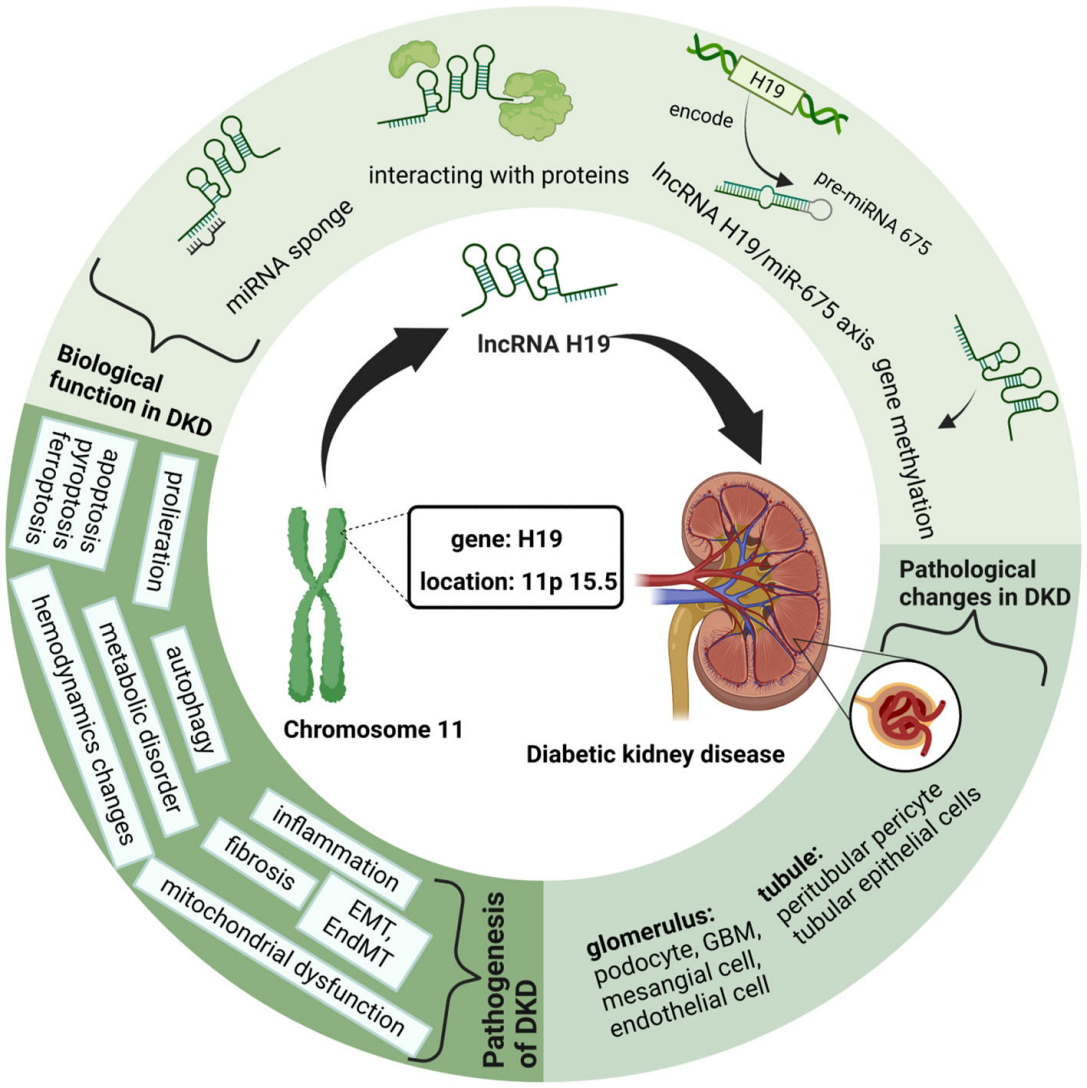
Graphical Abstract: LncRNA H19 in Diabetic kidney diseases. This figure is created with BioRender.com, and the authors have been granted a license to use the BioRender content.

Among them, lncRNA H19, the first imprinted and maternally expressed gene to be discovered in eukaryotes (18), is located close to the telomeric region of chromosome 11p15.5 and forms an imprinted domain with the gene insulin-like growth factor 2(IGF2), which is expressed from the paternal allele (19). Notably, during the development of kidney embryos, lncRNA H19 is extensively expressed in the ureteric bud branches and epithelial components of the metanephros, whereas its expression dramatically declines and fades in the postnatal period (20). Occasionally, lncRNA H19 is re-expressed during the activation of tumors, tissue repair, and intracellular stress, exhibiting its significance in aging, cancer, and liver diseases (21–23). Similarly, researchers found that H19 is upregulated in patients with DKD compared to that in the control group, and further studies have suggested the molecular mechanism of lncRNA H19 in regulating different processes of DKD (24). Therefore, in this review, we outline the latest developments in lncRNA H19 for modulating the pathophysiological processes of DKD and propose prospective future treatment strategies for DKD (**Figure 1**).

## 2 ‘Sophisticated’ structure changes in DKD

### 2.1 Key changes in the diabetic glomerulus

Hyperglycemia affects nearly all renal components, including the glomeruli, tubules, and interstitium. However, the glomerulus is traditionally classified as the first step in DKD. The Renal Pathology Society established four categories of glomerular lesions in 2010: Class I, glomerular basement membrane (GBM) thickening; Class II, mesangial expansion; Class III, nodular sclerosis (Kimmelstiel-Wilson lesions); and Class IV, advanced diabetic glomerulosclerosis (25).

GBM thicking, glomerular hypertrophy, and glomerular filtration barrier (GFB) deficiencies are early signs of glomerular alterations (26). Among these, the initial and most typical change in glomeruli is GBM thickening, which is also a hallmark of podocytes and glomerular endothelial cells (GECs) dysfunction (27). GBM is composed of laminins (LM), nidogens, collagen IV (Col. IV), and heparan sulfate proteoglycans, all of which are required for glomerular capillary wall formation and kidney filtration (28). Along with GBM, GECs and podocytes are the other two main constituents of GFB; when they are in a dysfunctional state, they contribute to albuminuria and a decrease in GFR (29, 30).

The dominant alteration in the glomerulus is glomerular podocyte dysfunction or podocytopathy, which is marked by podocyte loss, cellular hypertrophy, and foot process effacement (31, 32). Reactive oxygen species (ROS) produced during hyperglycemia trigger podocyte detachment and apoptosis, ultimately resulting in podocyte loss (33). Due to the limited potential for podocyte regeneration, high glucose levels induce an adaptive response in the form of podocyte hypertrophy, which aims to maintain glomerular integrity and reduce glomerulosclerosis (34–36). As a result, both podocyte hypertrophy and deletion are responsible for the podocyte foot process effacement (37).

Additional glomerular lesions include deletion of endothelial fenestrations and expansion of the mesangial matrix. The negatively charged glycocalyx decorated on fenestrated endothelial cells is essential for maintaining fluidic equilibrium and controlling vascular permeability, as well as preventing blood cells from attaching to the vascular wall (38, 39). Hence, elimination of specific glycocalyx components such as keratan sulfate, chondroitin sulfate, dermatan sulfate, and hyaluronic acid, enhances the endothelial permeability and triggers microalbuminuria (40, 41). Similarly, the continual reduction in the surface area of the fenestrated endothelium is an outcome of the death and pyroptosis of podocytes brought on by high glucose levels (42, 43).

Furthermore, hyperglycemia accelerates matrix expansion and alterations in glomerular mesangial cells (GMCs), which are the central stalk of the glomerulus making up 30-40% of all glomerular cells and role as a functional unit with endothelial cells and podocytes (44, 45). The mechanism of GMCs alterations in DKD is convoluted. It is commonly acknowledged that elevated blood glucose promotes the formation of the extracellular matrix (ECM), which is connected to altered GBM remnants, and stimulates pro-apoptosis or pro-proliferation signaling, which boosts apoptosis, proliferation, and hypertrophy in mesangial cells, all of which ultimately leads to proteinuria and glomerular hyperfiltration (46–50). Notably, mesangial cells increase the levels of Col. IV, plasminogen activator inhibitor 1 (PAI-1), and fibronectin (FN) to induce glomerular fibrosis (51). Additionally, endothelial-to-mesenchymal transition (EndMT) and epithelial-to-mesenchymal transition (EMT) are involved in renal fibrosis (52, 53).

### 2.2 Renal tubular dysfunction and fibrogenesis

**Figure 2:**
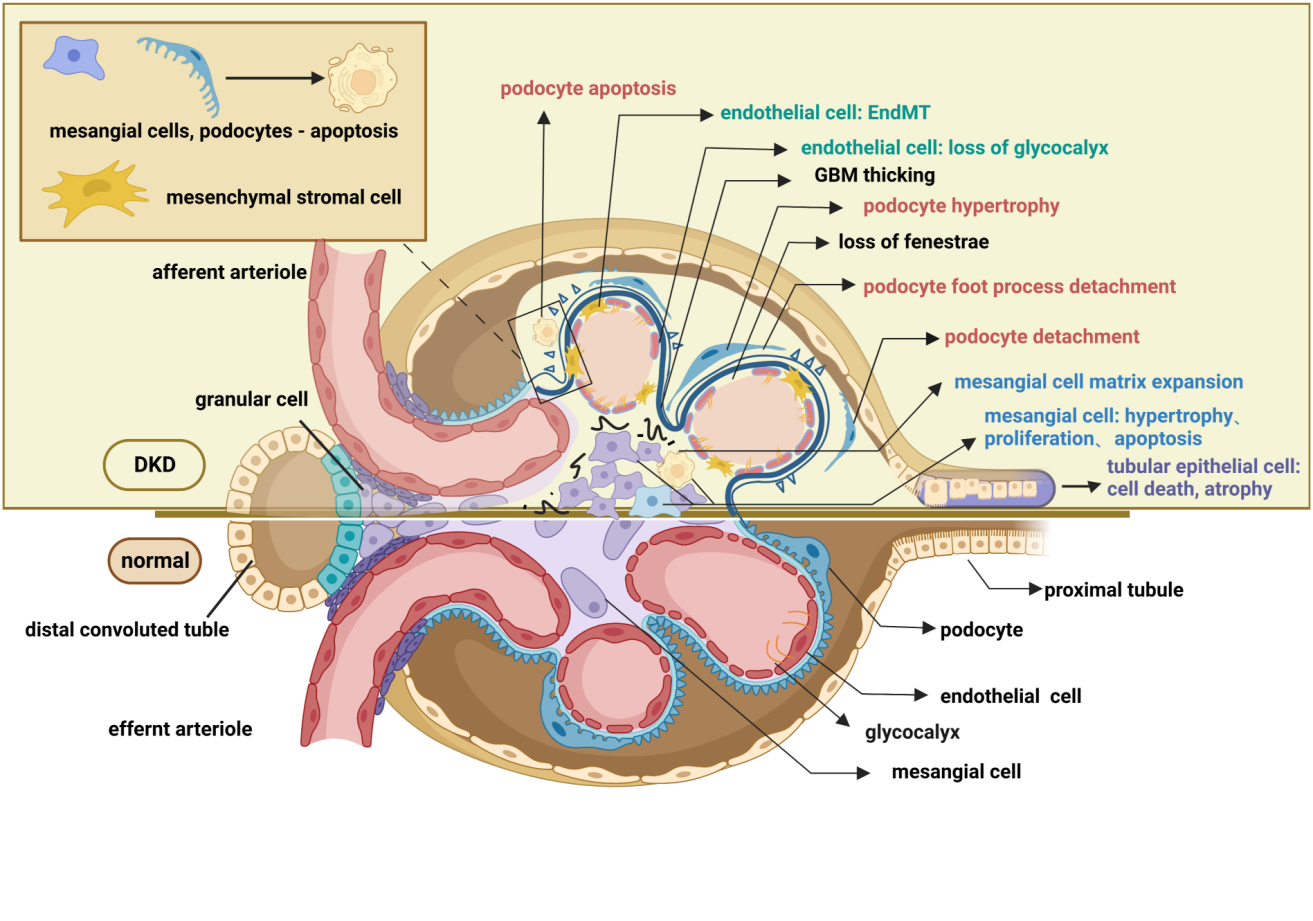
Pathological changes in the glomerulus and renal tubule of DKD. The pathological changes of the glomerulus mainly involve glomerular endothelial cells, mesangial cells, glomerular basement membrane, and podocytes. Endothelial cell changes include loss of glycocalyx, endothelial-mesenchymal transformation, apoptosis, and pyroptosis. Changes in podocyte include podocyte apoptosis, podocyte hypertrophy, podocyte detachment, podocyte loss, and podocyte foot process effacement. Mesangial cell changes include proliferation, hypertrophy, and apoptosis. Pathological changes in the renal tubules include tubular epithelial cell apoptosis ferroptosis, pyroptosis, autophagy, epithelial-mesenchymal transformation, and peritubular pericyte migration. These lesions, as well as the thickening of the basement membrane and the accumulation of extracellular matrix, contribute to the exacerbation of fibrosis in diabetic kidneys. This figure is created with BioRender.com, and the authors have been granted a license to use the BioRender content.

In contrast to careful studies on glomerular degeneration over the past few years, renal tubule interstitial impairment in DKD has largely been disregarded. Currently, the particular role of the renal tubule is of great interest in scientific research. Situated in the outer layer of the renal tubule, renal tubular epithelial cells (TECs) reabsorb chemicals such as amino acids and glucose from the urine, which is crucial for preserving the glomerulotubular balance. Additionally, TECs also influence the glomerular filtration rate by regulating concentrations of ions like Na+ and Cl-; this procedure is known as tubule-glomerular feedback (54, 55). In the early stages of DKD, renal tubular cells exhibit adaptive hypertrophy and elongation to maintain the glomerulotubular balance in response to glomerular hyperfiltration (56, 57). Subsequent histological alterations in the tubulointerstitium include tubular atrophy, peritubular capillary rarefaction, and inflammation, all of which contribute to the development of renal fibrosis (9, 58). Cell death functions as the main mechanism of renal tubular atrophy which is manifested by caspase-1-dependent pyroptosis, transforming growth factor-β(TGF-β1)-dependent ferroptosis, and caspase-3-dependent apoptosis (59–61). Moreover, increased diabetes-related inflammation encourages EMT in tubular epithelial cells and peritubular pericyte migration, both of which induce tubulointerstitial fibrosis (62, 63). Microvascular rarefaction refers to the peritubular pericyte moving out of the capillary and into the interstitial space (64), whereas EMT is marked by the acquisition of the mesenchymal markers smooth muscle actin (α-SMA) and the loss of the intracellular adhesion protein E-cadherin (65). As mentioned above, the hallmark pathological renal changes in DKD, such as GBM thickening, podocytopathy, loss of glomerular endothelium fenestrations, mesangial matrix expansion, and tubulointerstitial fibrosis, have all been well documented (**Figure 2**).

## 3 Pathogenesis of diabetic kidney disease

Considering that the underlying cellular processes resulting in the aforementioned histological presentations are considerably complex, continued attention to the pathophysiology of DKD is required. There is general agreement that critical metabolic derangements affect kidney hemodynamics and encourage immune dysregulation in early diabetes (66). Owing to the direct relevance of metabolic disorders, dyslipidemia, and intracellular stress, there is an increasing level of ROS, advanced glycation end products (AGEs), and lipid deposition (67–69). Moreover, intracellular stress includes hypoxia, endoplasmic reticulum (ER) stress, and OS (70), which generates ROS such as peroxides, superoxide, and hydroxyl radicals (71), which are linked to podocyte damage, focal segmental glomerulosclerosis (FSGS), and tubulointerstitial fibrosis (72). Activation of the RAAS is likely correlated with hemodynamic mechanisms, such as glomerular hyperfiltration and hypertension (73–75), which have long been known to be responsible for the initiation and propagation of DKD (76). Additionally, interactions between metabolic disorders and hemodynamic abnormalities lead to immunological dysfunction including inflammation and renal fibrosis by triggering the production of pro-inflammatory and pro-fibrotic mediators (77–79). Moreover, emerging evidence indicates that autophagy dysregulation, mitochondrial dysfunction, apoptosis, pyroptosis, and ferroptosis contribute to renal damage in DKD (78, 80–83).

## 4 LncRNA biology

LncRNAs are abundant as functional units in eukaryotes, performing precise expression patterns in their various subcellular localizations, which has recently been the focus of intense debate. Numerous lncRNAs are limited to and abundant in the nucleus, governing transcriptional programs via chromatin remodeling and interactions in trans or cis (84). However, some are present in the cytoplasm, where they mediate signal transduction pathways, translational programs, or post-transcriptional genetic regulation, whereas others are present in the mitochondria and ER, where their functions remain still obscure (85).

Depending on their roles, lncRNAs are commonly categorized as signals, decoys, scaffolds, and guides molecules that engage in diverse biological processes that affect gene expression, including epigenetic, transcriptional, post-transcriptional, translational, and post-translational regulation (16, 86). LncRNAs unlock the capabilities of epigenetic gene regulation by affecting histone modifications close to gene transcriptional start sites, boosting methylation of the gene promoter, and encouraging chromatin remodeling (87–89). Indeed, lncRNAs actively participate in transcriptional regulation by either trapping and regulating transcription factors (TF) or functioning as active enhancers to stimulate or suppress transcription (90–92). In addition to their roles in epigenetic and transcriptional regulation, lncRNAs also handle post-transcriptional regulation through several approaches, including the formation of specific lncRNA-protein complexes (lncRNPs) that operate as competitive endogenous RNAs (ceRNAs) or “sponges” of microRNAs (miRNAs), thereby regulating alternative splicing, mRNA degradation and diminishing mRNA stability (93–97). Furthermore, lncRNAs regulate several other aspects of gene expression, such as the disruption of translational processes by binding to eukaryotic translation initiation factor 4 gamma 2 (EIF4G2) and post-translational regulation via protein interactions (98, 99).

## 5 Classical lncRNAs in DKD

Thus, understanding lncRNAs in eukaryotes has broadened our understanding of the role of lncRNAs in regulating gene expression and other biological processes. Reports on the interactions between lncRNAs and DKD have emerged in recent years, suggesting their essential roles in the pathogenesis of DKD. LncRNAs regulate many crucial factors linked to the DKD progression in different cell masses (100).

### 5.1 Podocyte injury: autophagy, apoptosis, mitochondrial dysfunction

For example, a transcript called lncRNA AK044604 (Risa) is situated very close to the sirt1 gene, which is recognized as a modulator of autophagy and insulin sensitivity, contributing to the onset of DKD through the regulation of podocyte autophagy and the thickness of GBM (101). Furthermore, decreased levels of the lncRNA taurine-upregulated gene 1(Tug1) are observed in podocytes exposed to high glucose (HG) stimuli, exhibiting a reno-protective role in DKD through the lncRNA Tug1/PGC1α axis, which is essential for improving mitochondrial function (102). In contrast, lncRNA maternally expressed gene 3 (Meg3) is upregulated under the HG conditions, thereby increasing dynamin-related protein 1 (Drp1) expression and its phosphorylation or translocation, which triggers mitochondrial fission and podocyte damage in diabetic mice induced by streptozotocin (STZ) (103). Similarly, lncRNA PVT1 is up-regulated in DKD patients, and *in vitro* experiments explain that it encourages the recruitment of histone 3 lysine 27 trimethylation (H3K27me3) in the forkhead box A1(FOXA1) promoter region by recruiting enhancer of zeste homolog 2 (EZH2), which lessens FOXA1 expression, thereby increasing apoptosis and damage levels in podocytes (104).

### 5.2 Tubular epithelial cells: EMT and fibrosis

Overexpression of lncRNA NEAT1 is observed in the TECs which further promotes EMT and fibrosis through the ERK1/2 pathway resulting in the accumulation of critical cytokines like connective tissue growth factor (CTGF), TGF-β, vimentin, and α-SMA (105). Additionally, under the treatment of TGF-β1, cytoplasmic lncRNA growth arrest-specific 5 (GAS5) expression is significantly increased in the human proximal tubule cell line (HK-2) and exerts its biological effects by serving as a sponge for the miR-96-5p and enhancing the formation of fibronectin to exacerbate renal fibrosis (106). Furthermore, in the AGE-treated GECs and TECs, the expression of lncRNA Erbb4-IR is dramatically elevated in a Smad3-dependent way which can boost the production of Col. I and Col. IV, as well as worsen renal fibrosis by sponging miR-29b (107).

### 5.3 Mesangial cells: proliferation and ECM accumulation

LncRNA NR_033515, competitively binding to miR-743b-5p, is engaged in fibrosis, EMT, and proliferation in DKD as evidenced by the rise in fibrogenic proteins and epithelial cell markers such P38, α-SMA, FN, E-cadherin, as well as mesenchymal marker Vimentin (108). Moreover, in the HG-treated mesangial cells, lncRNA cyclin-dependent kinase inhibitor 2B antisense RNA 1(CDKN2B-AS1) is significantly elevated which binds to miR-424-5p to stimulate the production of high mobility group AT-hook 2(HMGA2). The levels of the proteins linked with PI3K/AKT signaling are increased through the DKN2B-AS1/miR-424-5p/HMGA2 axis, together with the stimulation of cell proliferation and ECM buildup (109).

## 6 Biological functions of lncRNA H19 in DKD pathogenesis

In addition to the aforementioned lncRNAs, H19 has received an avalanche of interest from researchers because of its unique biological characteristics. Because of its differential expression during the endocrine progenitor stage of pancreatic-islet development, H19 is crucial for pancreatic-islet development and function (110). LncRNA H19 is highly expressed under hyperglycemia and serves as a crucial regulator in many pathophysiological processes of DKD, including the inflammatory response, EMT, cellular proliferation, apoptosis, autophagy, and fibrosis. We outline the following four modes of action of lncRNA H19 in modulating DKD: its role as a miRNA sponge, role in gene methylation, role as a precursor of miR-675, and role in interacting with protein. Additionally, we illustrated the regulatory mechanism of lncRNA H19 in different renal cells (**Table 1**).

**Table 1:** The mechanism of lncRNA H19 in different renal cells in diabetic kidney diseases.

Expression	Samples	Targets	Functions	References
up	Mesangial cells	miR-143-3p, IL-6, TNF-α, TGF-β1, Col. IV, and FN	Inflammation, Proliferation, Fibrosis	(111)
up	Serum	IL-6, TNF-α	Fibrosis	(112, 113)
up	STZ-mice, podocyte	miR-29a, TGFβ, Smad3, FSP-1, CD31	Fibrosis, EndMT	(114, 115)
up	HK-2 cells	miR-17, α-SMA, FN, Col. IV, and Col. I	Fibrosis, EMT	(116)
up	Mesangial cell	miR-129, HMGB1, Nrf2	Ferroptosis	(117)
up	podocytes	NLRP3	Pyroptosis	(118)
up	podocytes	ATG7	Autophagy	(119)
up	serum	Beclin-1	Autophagy	(120)
up	Tubular epithelial cells	DIARS3, mTOR	Autophagy	(121)
up	CIHP‐1, HEK 293 cells	miR-675, VDR, EGR1	EMT, Fibrosis,	(122)
up	Diabetic mice	MFN-2	Mitochondrial dysfunction	(123)
up	DKD rats, glomerular endothelial cells	Akt/eNOS	GEC damage	(124–126)

### 6.1 Role as a miRNA sponge

**Figure 3:**
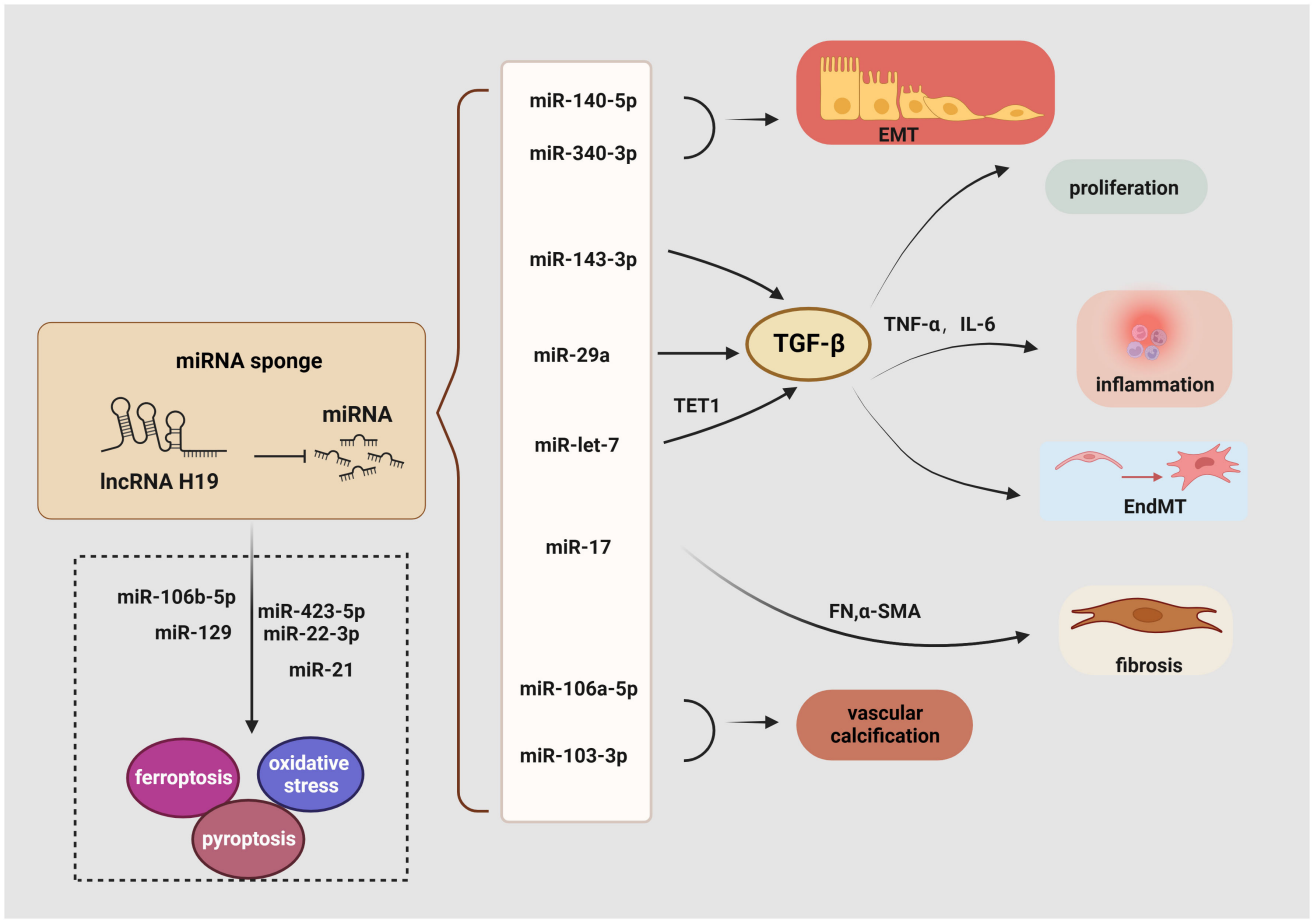
Role of lncRNA H19 as a miRNA sponge in DKD. LncRNA H19 could sponge miRNA inhibits its expression and engage in regulating the diverse pathogenesis of DKD, including EMT, EndMT, inflammation, proliferation, oxidative stress, ferroptosis, pyroptosis, autophagy, and fibrosis. →: promote, —: inhibit. This figure is created with BioRender.com, and the authors have been granted a license to use the BioRender content.

LncRNA H19 serves as a miRNA sponge to regulate EndMT, proliferation, inflammation, EMT, fibrosis, oxidative stress, ferroptosis, and pyroptosis (**Figure 3**). For example, a considerable increase in lncRNA H19 expression has been observed in mouse mesangial cells under HG conditions. Then, the luciferase reporter assay demonstrated that lncRNA H19 binds to miR-143-3p to boost the production of inflammatory molecules interleukin-6 (IL-6) and TNF-α as well as pro-fibrogenic molecules like TGF-β1, Col. IV, and FN, thereby advancing the inflammation and proliferation of GMCs (111). Similarly, in the serum of clinical patients with CKD, lncRNA H19 was positively linked with the expression of TNF-α and IL-6, the latter served as a risk factor for DKD (112, 113). Consistent with these findings, lncRNA H19 was up-regulated in both the STZ-induced animal model and the TGF-β2-induced human microvascular endothelial cells (HMVECs), blocking miR-29a expression to significantly increase the activity of the TGF-β/Smad3 pathway (114). In addition, miR-29a promotes nephrin acetylation to reduce the effects of hyperglycemia on podocytes (115). Further research has revealed that in diabetic kidneys, lncRNA H19 knockdown inhibits EndMT by upregulating the expression of mesothelial cell marker FSP-1 and downregulating the endothelial marker CD31 (114).

Another recent study demonstrated a favorable correlation between lncRNA H19 and renal fibrosis. Using TGF-β-induced HK-2 cells, StarBase 2.0 to identify miRNA recognition sites associated with lncRNA H19 during renal fibrosis, including miR-20, miR-93, miR-106, miR-18, and miR-17. It was shown that lncRNA H19 can function as an endogenous RNA to decrease miR-17 levels and boost the production of α-SMA, FN, Col. IV, and Col. I but reduce E-cadherin in unilateral ureteral obstruction mice. These findings collectively show that lncRNA H19 exacerbates renal fibrosis in DKD via the miR-17/fibronectin regulatory networks (116). Moreover, by serving as a miRNA sponge for let7, lncRNA H19 positively controlled the production of ten-eleven translocation (TET1), which in turn influences the epigenetic regulation of upstream TGF signaling genes including thrombospondin 1 (TSP1) and TGF‐β receptor 2 (TGFBR2). This increased the phosphorylation of the TGF signaling intermediate SMAD2 and the overexpression of the EndMT markers FN, vimentin, and smooth muscle 22 alpha (SM22‐α). Based on these findings, it suggests that the lncRNA H19/TET1 axis may contribute to phenotypic alterations during DKD progression (127). Besides, lncRNA H19 activates the PI3K/AKT pathway and regulates tyrosine 3-monooxygenase/tryptophan 5-monooxygenase activation protein zeta (YWHAZ) expression to promote EMT by working as an endogenous sponge for miR-340-3p and miR-140-5p (128, 129). Similarly, binding to and suppressing miRNAs like miR-138 and miR-148a, lncRNA H19 promoted the EMT markers along with stabilizing TGF-β through lncRNA H19/miR-138/SOX4 and lncRNA H19/miR-148a/ubiquitin-specific protease 4 (USP4) axes, which may further result in fibrosis (130, 131). Through sponging miR-106a-5p or miR-103-3p in a Runx2 dependent way, elevation of H19 contributes to increased vascular calcification (VC) in the kidney (132, 133).

Ferroptosis is another potential target of lncRNA H19 in DKD. Previous studies revealed that curcumin therapy markedly decreases the production of the lncRNA H19 in lung cancer cells to induce ferroptosis. Mechanistically, lncRNA H19, which works as a rival endogenous RNA bounding to miR-19b-3p, suppresses ferroptosis as shown by the increased transcriptional activity of ferritin heavy chain 1 (FTH1), a gene that is both an endogenous target of miR-19b-3p and a depressor of ferroptosis (134). Additionally, lncRNA H19 knockdown increased cell division and decreased ferroptosis in brain microvascular endothelial cells (BMVECs) by modulating the miR-106b-5p/acyl-CoA synthetase long-chain family member 4(ACSL4) axis (135). Intriguingly, an increase in the tubular pro-ferroptosis gene ACSL4 was linked to the renal function of acute kidney tubular injury patients, which triggered ferroptosis in TECs (136). Furthermore, lncRNA H19 has been shown to serve as a sponge for miR-129 (137), and, positively regulate ion of high-mobility group box 1 (HMGB1) which modulates oxidative stress and hyperglycemia-induced ferroptosis in mesangial cells through the nuclear-related factor 2 (Nrf2) pathway (117).

Given the crucial role of autophagy and pyroptosis in the progression of DKD, the regulatory relationship between lncRNA H19 and regulated cell death (RCD) cannot be neglected. Previous research has illustrated that lncRNA H19 also serves as a sponge for miR-22-3p and miR-423-5p to boost the activity of the Nod-like receptor family pyrin domain-containing 3 (NLRP3) (138, 139), a marker of pyroptosis and a factor in podocyte damage, thereby encouraging pyroptosis (118). Besides targeting miR-423-5p and miR-22-3p, lncRNA H19 also exhibits negative effects on miR-21, whereas the reduction of miR-21 enhances the expression of programmed cell death 4 (PDCD4), forming a competing endogenous RNA network (ceRNET) that significantly promotes an imbalance in the NLRP3/6 inflammasome, leading to pyroptosis (140). Another fascinating finding suggests that lncRNA H19 reverses mitochondrial damage and cell growth inhibition by sponging miR-93-5p under the condition of lipopolysaccharide (LPS) (141). Similarly, increased levels of lncRNA H19 trigger cellular autophagy via the lncRNA H19/miR-143/autophagy-related protein 7 (ATG7) signaling axis (142), while ATG7 is a hallmark of podocyte autophagy (119).

**Figure 4:**
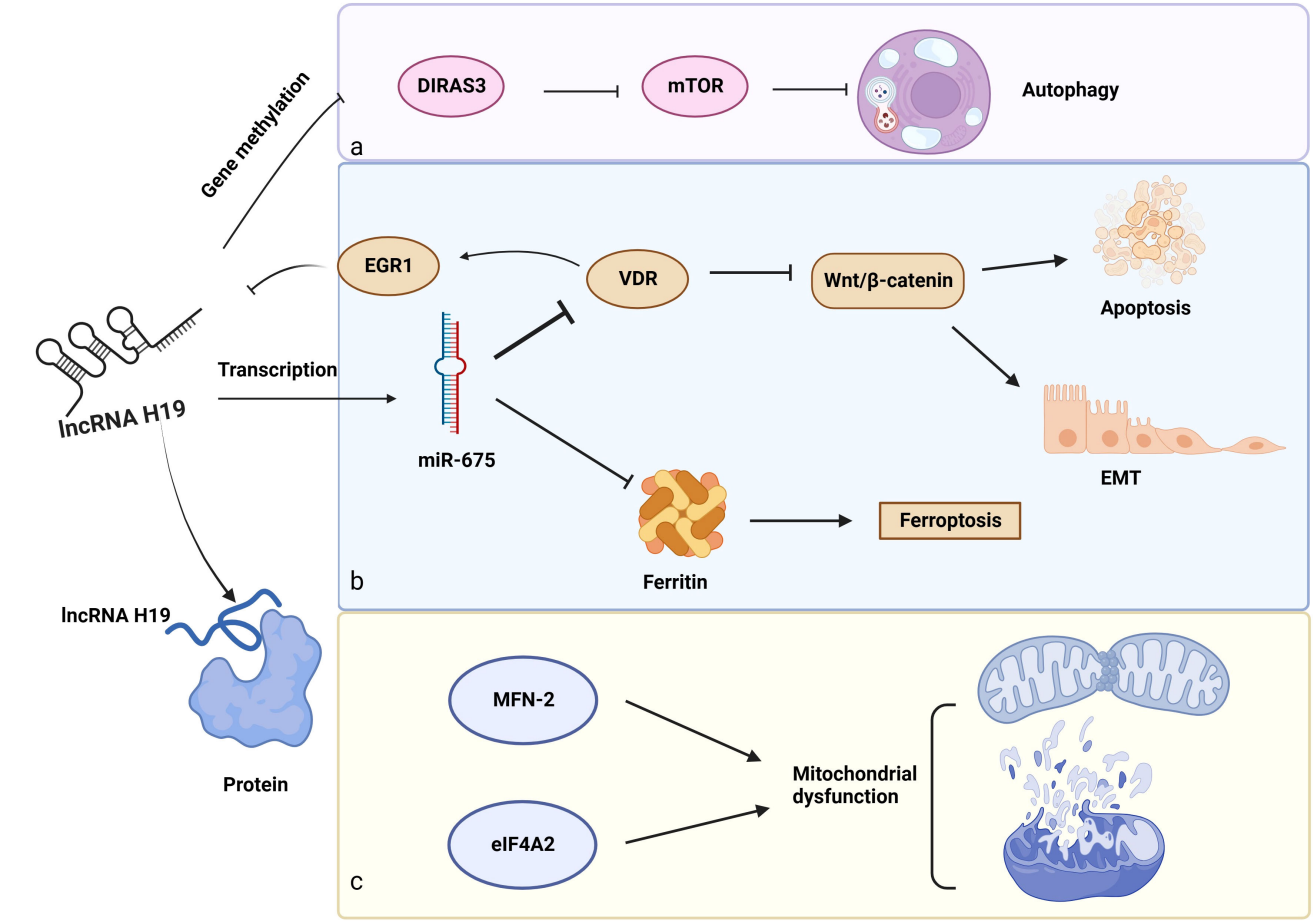
Other biological functions of lncRNA H19 in DKD. a: Gene methylation: LncRNA H19 regulates gene methylation of DIRAS3 to inhibit autophagy. b: Role of the lncRNA H19/miR-675 axis in DKD, c: LncRNA H19 interacts with proteins (MFN-2 and eIF4A2) to induce mitochondrial dysfunction. →: promote, —: inhibit. This figure is created with BioRender.com, and the authors have been granted a license to use the BioRender content.

Apart from a miRNA sponge, lncRNA H19 exhibits its regulating effects in gene methylation, protein interaction, and transcription of small RNAs (**Figure 4**).

### 6.2 Role in gene methylation

In addition to acting as a miRNA sponge, lncRNA H19 influences the gene methylation of downstream cytokines. These findings suggest that lncRNA H19 binds to and inhibits the enzyme S-adenosylhomocysteine hydrolase (SAHH), resulting in the build-up of SAH, which prevents the transcription factor DNA methyltransferase 3 B (DNMT3B) from methylating the Beclin1 promoter. As a consequence, lncRNA H19 encouraged Beclin1 transcription to trigger autophagy (143). However, the serum of patients with DKD has lower levels of Beclin-1, pointing to the probable involvement of lncRNA H19 and autophagy in this disease (120). Aplasia Ras homolog member I (DIRAS3), which is extensively expressed in epithelial cells, encodes a small GTP-binding protein (121). Interestingly, lncRNA H19 decreased the amounts of DIRAS3 and increased the mTOR-related proteins to inhibit podocyte autophagy (144), however, a high level of podocyte autophagy is essential to maintain renal homeostasis (145). Shensu IV, a well-known Chinese prescription, promotes lncRNA H19/DIRAS3-regulated autophagy to prevent kidney injuries in DKD (144).

### 6.3 Role as a precursor of small RNAs: lncRNA H19/miR-675 axis

Regarding the unique structures of miR-675 and H19, scientists have revealed some interesting findings. MiR-675, which is embedded in the first exon of H19 gene (146), is activated in response to increased lncRNA H19 synthesis, indicating that lncRNA H19 modulated the transcription of miR-675. Luciferase reporter assay further confirmed this combination. What’s more, 3′ UTR of vitamin D receptor (VDR) contains a complementary sequence of miR-675, providing a regulatory possibility for miR-675. The VDR is negatively linked to early growth response protein 1 (EGR1), a transcription factor of lncRNA H19. Furthermore, EGR1 stimulated the Wnt/β-catenin pathway, the classical signal of EMT, to accelerate renal fibrosis. These observations suggest a negative feedback loop for lncRNA H19/miR-675/EGR1 during the progression of DKD (24, 122). Notably, there might be a similar function of lncRNA H19/miR-675 in DKD because of its role in regulating EMT in hepatocytes, breast cells, and skin squamous cells (147–149), as well as its inhibitory effect on iron-stored protein ferritin (FHC), which is necessary to maintain iron metabolism in the kidney (150).

### 6.4 Role in interacting with protein

LncRNA H19 also influences downstream protein expression via direct binding. By binding to heterogeneous nuclear ribonucleoprotein A2B1(hnRNPA2B1), lncRNA H19 maintains and increases the expression of Raf-1 to activate the Raf-ERK signaling linked to EMT (151). In addition, overexpression of lncRNA H19 prevented Pink1 mRNA from binding to eukaryotic translation initiation factor 4A, isoform 2 (eIF4A2), blocking the translation of Pink1 and reducing mitophagy through the Pink1/Parkin pathway (152). Furthermore, in diabetic mice, H19 interacts with mitofusin-2 (MFN-2) mRNA. MFN-2 is a dynamin GTPase found on the outer mitochondrial membrane that is encoded by nuclear genes and is responsible for outer mitochondrial membrane fusion (123).

### 6.5 Role in other regulating factors related to DKD

However, some studies did not explain the specific working patterns of lncRNA H19 in DKD, leading to the following conclusions. One study showed that lncRNA H19 is highly expressed in both DKD rats and rat glomerular endothelial cells (rGEnCs). Further experiments demonstrated that lncRNA H19 knockdown reduced the GBM and ameliorated GEC impairment, as indicated by the high levels of the principal glycocalyx components WGA and Syndecan-1, as well as the essential tight junction proteins ZO-1 and Occludin. Additional research has demonstrated that lncRNA H19 has a deleterious effect on DKD by blocking Akt/eNOS signaling, which is essential for podocyte and GEC damage (124–126). According to a recent study, phospholipid hydroperoxide glutathione peroxidase (GPX4), a factor that negatively regulates ferroptosis, is favorably linked to lncRNA H19 levels during spontaneous abortion (153). Similarly, renal biopsy samples from patients with DKD show lower GPX4 expression than those from healthy controls, suggesting an independent predictor of ESKD development (154). These discoveries will make it possible to understand the relationships between lncRNA H19 and GPX4, which will help choose therapeutic targets to control ferroptosis and manage DKD progression.

In other disease models, lncRNA H19 plays a role in collective pathogenesis, providing more possibilities for the role of lncRNA H19 in DKD. For instance, lncRNA H19 promoted oxidative stress and released inflammatory factors IL-6, IL-1β, and TNF- by stimulating the NF-kB pathway in intracerebral hemorrhage (ICH) rats (155). Additionally, lncRNA H19 might encourage the translocation of β-catenin into the nucleus and trigger Wnt/β-catenin signaling, leading to EMT (156). In hepatocytes induced by IL-22, it is interesting to note that an elevation in lncRNA H19 influenced the expression of t AMPK and AKT proteins, which are upstream regulators of mTOR signaling, ultimately preserving mitochondrial function and integrity by activating the AMPK/AKT/mTOR axis (157). Besides, it has been proven that lncRNA H19 lowered the formation of ROS and lessened mitochondrial damage by suppressing the NF-kB activation driven on by ox-LDL (158).

## 7 Role of lncRNA H19 in nondiabetic kidney disease

Nondiabetic kidney disease (NDKD) refers to kidney diseases unrelated to diabetes, including glomerulonephritis, acute kidney injury (AKI), focal segmental glomerulosclerosis, and minimal change disease. Nevertheless, renal biopsy in diabetic patients with chronic kidney diseases may indicate the following three possibilities: DKD, NDKD, or a mixture of both DKD and NDKD (159). Therefore, additional investigations of lncRNA H19 in NDKD are helpful to fully understand its role in DKD.

DKD patients are more susceptible to AKI, which has been shown to deteriorate kidney function and is challenging to recover from when it occurs in DKD (160). In AKI model mice, the elevated expression of lncRNA H19 has been shown to stimulate the synthesis of Wnt and β-catenin through sponging miR-196a-5p, promoting Wnt/β-catenin signaling pathway, which in turn promotes renal fibrosis (161). Meanwhile, another study verifies that lncRNA H19 regulates the apoptosis, proliferation, and inflammation of TECs in a miR-130 manner. The pro-apoptotic proteins are elevated whereas the anti-apoptotic protein Bcl-2 is decreased in HK-2 cells due to the lncRNA H19/miR-130a axis. Additionally, the lncRNA H19/miR-130a/BCL2L11 axis stimulates the production of IL-6, IL-1, and TNF-α while suppressing IL-10, resulting in inflammation in the TECs (162). Notably, inconsistent outcomes are observed in the renal adeno-associated virus 2 (AAV2)-mediated mouse model. LncRNA H19 is observed to upregulate in the kidney biopsies of AKI patients. *In vivo* experiments then confirm that lncRNA H19, which sponges miR-30a-5p, could attenuate apoptosis and inflammation while also stimulating pro-angiogenic signaling, suggesting a protective role against kidney injury. It proves that capillary density and tubular epithelial integrity can be preserved in the lncRNA H19/miR-30a-5p axis (163). Altogether, these findings imply that lncRNA H19 might be crucial in AKI.

Researchers found that lncRNA H19 was overexpression in the glyoxylate-induced Calcium oxalate (CaOx) nephrocalcinosis mouse models. Their additional findings that lncRNA H19 accelerates renal epithelial cell injury in a miR-216b-5p manner, thereby up-regulating the expression of HMGB1, another target of miR-216b-5p that is negatively linked with it. Then HMGB1 interacted with toll-like receptor 4 (TLR4) to promote the transcription and expression of several chemokines and proinflammatory cytokines like IL-6, IL-1β, and TNF-α via NF-kB or NADPH oxidase ways (164). It is recognized that TLRs are endogenous danger-associated molecular patterns produced in DM that allow for the stimulation of the NF-kB signaling to trigger a sterile tubulointerstitial inflammatory response (77). These results collectively revealed a possible involvement for the lncRNA H19/mir-216b-5p/HMGB1 axis in the pathology of TEC injury through the modulation of OS and inflammatory response (164). It was recently discovered in nephroblastoma cells that lncRNA H19 significantly influences the miR-675/TGFBI axis to enhance proliferation and prevent apoptosis (165). Intriguingly, it has been shown that IGF2 and lncRNA H19 compete for the same binding enhancer, and the lncRNA H19-IGF2 imprinted gene area is likely linked to an increased risk of having compromised renal function (IRF) (166). Otherwise, renal clear cell carcinoma, Wilms tumors, and renal cell carcinoma are all influenced by the interaction between lncRNA H19 and IGF2 (167–169).

## 8 Conclusion and perspectives

This review focuses on the primary pathogenic alterations and mechanisms of DKD, as well as the functions of lncRNAs in DKD, highlighting lncRNA H19. Substantial evidence supports the essential role of lncRNA H19 in the physiology and pathology of the diabetic kidney. As a result, we outlined how lncRNA H19 might affect the phenotypes associated with DKD, including inflammation, oxidative stress, autophagy, mitochondrial dysfunction, EMT, EndMT, ECM buildup, and fibrosis. We divided the mechanisms of lncRNA H19 in controlling DKD into four groups at the molecular level: regulation of gene methylation, protein interactions, miRNA sponges, and small RNA precursors. Among these, we find that serving as miRNA sponges is more common for lncRNA H19, which sponge miRNA to regulate downstream factors to modulate fibrosis-related progressions and hastens the development of DKD. Previous research revealed that lncRNA H19 is an independent risk factor for T2DM. Increased renal indicators (urea, creatinine, and UACR) and nephropathy are more frequently linked to H19 expression (170, 171). In addition, the discovery that metformin, a first-line therapy for type 2 diabetes, might lessen kidney damage through downregulating lncRNA H19, and therefore DKD, offers another direction for our investigation of the potential of metformin for treating DKD. Despite these developments, many unresolved problems persist. Drugs targeting lncRNA H19 are limited. We can focus on some Chinese traditional medicine such as Shensu IV to deeply explore the mechanisms of lncRNA H19, providing novel therapy for DKD patients. Hence, in-depth research on lncRNA H19 is required. More mechanistic and translational studies with tissue- and cell-type-specific H19−/− animal models are needed. Additionally, studies are required to validate preclinical findings in human samples and to examine the effects of compounds that modulate lncRNA H19 in DKD clinical trials. In the future, we propose a hypothesis that we can identify a functional motif of H19 and deliver the functional motif to kidney cells to alleviate kidney injuries, which is similar to some lncRNA-targeted therapy in pathological cardiac hypertrophy and tumor disorders. Furthermore, given the imprinted properties of lncRNA H19, we may utilize its expression variations to investigate the prevalence of DKD in various sexes, offering novel approaches for illuminating the genetic landscape and the early detection of DKD. In a word, lncRNA H19 provides more possibilities for us to diagnose and treat DKD.

## Author contributions

QW wrote and revised the manuscript. FH critically revised the manuscript. All authors contributed to the article and approved the submitted version.
